# SARS-CoV-2 infects human brain organoids causing cell death and loss of synapses that can be rescued by treatment with Sofosbuvir

**DOI:** 10.1371/journal.pbio.3001845

**Published:** 2022-11-03

**Authors:** Pinar Mesci, Janaina S. de Souza, Laura Martin-Sancho, Angela Macia, Aurian Saleh, Xin Yin, Cedric Snethlage, Jason W. Adams, Simoni H. Avansini, Roberto H. Herai, Angels Almenar-Queralt, Yuan Pu, Ryan A. Szeto, Gabriela Goldberg, Patrick T. Bruck, Fabio Papes, Sumit K. Chanda, Alysson R. Muotri

**Affiliations:** 1 Department of Pediatrics/Rady Children’s Hospital-San Diego, Department of Cellular & Molecular Medicine, School of Medicine, University of California San Diego, La Jolla, California, United States of America; 2 Immunity and Pathogenesis Program, Infectious and Inflammatory Disease Center, Sanford Burnham Prebys Medical Discovery Institute, 10901 North Torrey Pines Road, La Jolla, California, United States of America; 3 Department of Medical Genetics, School of Medical Sciences, University of Campinas, Campinas, Sao Paulo, Brazil; 4 Experimental Multiuser Laboratory (LEM), Graduate Program in Health Sciences (PPGCS), School of Medicine, Pontifícia Universidade Católica do Paraná (PUCPR), Curitiba, Paraná, Brazil; 5 Research Department, Lico Kaesemodel Institute (ILK), Curitiba, Paraná, Brazil; 6 Department of Genetics, Evolution, Microbiology, and Immunology, Institute of Biology, University of Campinas (UNICAMP), Campinas, Sao Paulo, Brazil; 7 Center for Academic Research and Training in Anthropogeny (CARTA), University of California San Diego, La Jolla, California, United States of America; 8 Kavli Institute for Brain and Mind, University of California San Diego, La Jolla, California, United States of America; 9 Archealization Center, University of California San Diego, La Jolla, California, United States of America; UNITED KINGDOM

## Abstract

The Severe Acute Respiratory Syndrome Coronavirus 2 (SARS-CoV-2) is the causative agent of coronavirus disease 2019 (COVID-19), which was rapidly declared a pandemic by the World Health Organization (WHO). Early clinical symptomatology focused mainly on respiratory illnesses. However, a variety of neurological manifestations in both adults and newborns are now well-documented. To experimentally determine whether SARS-CoV-2 could replicate in and affect human brain cells, we infected iPSC-derived human brain organoids. Here, we show that SARS-CoV-2 can productively replicate and promote death of neural cells, including cortical neurons. This phenotype was accompanied by loss of excitatory synapses in neurons. Notably, we found that the U.S. Food and Drug Administration (FDA)-approved antiviral Sofosbuvir was able to inhibit SARS-CoV-2 replication and rescued these neuronal alterations in infected brain organoids. Given the urgent need for readily available antivirals, these results provide a cellular basis supporting repurposed antivirals as a strategic treatment to alleviate neurocytological defects that may underlie COVID-19- related neurological symptoms.

## Abstract 2

This study shows that SARS-CoV-2 virus can infect human brain cortical organoids, inducing cell death and synapse loss; application of Sofosbuvir, an FDA-approved antiviral, rescues these neuronal alterations in infected brain organoids.

Coronavirus disease 2019 (COVID-19), caused by the Severe Acute Respiratory Syndrome Coronavirus 2 (SARS-CoV-2), was declared a pandemic by the World Health Organization (WHO) on 11th March 2020. The outbreak quickly spread to 216 countries in a couple of months, with more than 626 million cases and more than 6.5 million confirmed deaths worldwide, as of this writing. Despite vaccines currently being distributed, new variants emerge rapidly, and thousands of new infections are still reported every day. Thus, fast-deployable and efficient antiviral therapies are urgently needed.

Early studies mainly focused on the respiratory component of COVID-19 disease. However, as more cases appeared, other COVID-19-related clinical manifestations began being reported. COVID-19 adult patients also presented with a variety of neurological symptoms, including stroke, hallucinations, epilepsy, encephalopathy, anosmia, and ageusia, suggesting that SARS-CoV-2 either directly or indirectly impacts the central nervous system (CNS) [1,2,3–10,11–18]. A recent publication conducted on *postmortem* tissues of COVID-19 patients found evidence that ciliated cells in the respiratory mucosa and that sustentacular cells (non-neuronal) in the olfactory mucosa are the main target cell types for SARS-CoV-2; routes through which olfactory sensory neurons could become affected [19]. A prospective study published aiming to determine the prevalence of the new neurological disorder in COVID-19 patients in the New York City metropolitan area reported that 13.5% of COVID-19 patients developed a neurological disorder [20]. Supporting this, another report found evidence that the S1 spike protein of SARS-CoV-2 was able to cross the blood-brain barrier in mice [21], suggesting that SARS-CoV-2 could infect the brain and potentially trigger long-term neurological manifestations. Finally, several reports now suggest that human brain cells are susceptible to SARS-CoV-2 virus infection as the virus was detected in cortical neurons of autopsies of patients who succumbed to COVID-19 [10,22–29]. Recent work has also shown that placentas from COVID-19-positive pregnant women display injury [30], and has reported cases of vertical transplacental transmission of SARS-CoV-2 to neonates born with neurological compromise [31]. A recent case study showed altered patterns of expression of entry factors for SARS-CoV-2 during critical developmental stages of the human embryo, including detection as early as the second week of pregnancy [32]. Together, these findings indicate a likelihood of vertical transmission of the virus to the fetus and the potential to affect fetal brain development [33]. Supporting this hypothesis, babies born to SARS-CoV-2-positive mothers have shown several inflammatory symptoms such as neonatal sepsis, rashes and eye infections while long-term impacts remain unknown [34].

Our laboratory previously revealed a causative link between the circulating Brazilian Zika virus and the severe microcephaly observed in babies born from infected mothers using human induced pluripotent stem cell (hiPSC)-derived brain organoids [35]. Human brain organoids are scaled-down, three-dimensional models of the brain that recapitulate several molecular and cellular aspects of human embryonic and fetal developmental stages [36]. At functional level, brain cortical functional organoids closely mimic the early stages of human neurodevelopment and organized cortical network development [37]. Here, we evaluated whether SARS-CoV-2 could infect human brain cells and viral impact on the developing human brain.

Angiotensin-converting enzyme-2 (ACE2) is a critical receptor for SARS-CoV-2, hence its expression has been used to predict the potential permissibility of different cell/tissue types [38,39]. Other factors, including TMPRSS2 (Transmembrane protease, serine-2), DPP4 (Dipeptidyl peptidase-4)/CD26, BSG (Basigin)/CD147, and NRP1 (Neuropilin-1) have been implicated in SARS-CoV-2 cellular entry [40–44]. To predict the ability of the CNS to support replication of SARS-CoV2, we used publicly available databases to screen for gene expression of these cellular factors [45–47] (see Methods). Using the TISSUES database (version 2.0), an integrative web resource on mammalian tissue expression that generates gene-tissue associations based on publicly available transcriptomic and proteomic data, we predicted protein and gene expression of these cellular factors in the human body (Fig 1A) [45]. This resource predicted that ACE2, TMPRSS2, CD147, and Neuropilin-1, but not CD26, are expressed in the brain with a high level of confidence (Fig 1A). From a transcriptional point of view, globally all these entry factors were expressed to a lesser extend in the CNS compared to other organs (S1A Fig). Within the CNS, ACE2, TMPRSS2, and DPP4/CD26 were lowly expressed in all brain regions studied, while NRP1 was highly expressed in the hippocampus and the cerebral cortex (S1B and S1C Fig). Finally, BSG/CD147 were found to be the highest expressed genes in all brain regions studied (S1C Fig).

**Fig 1:**
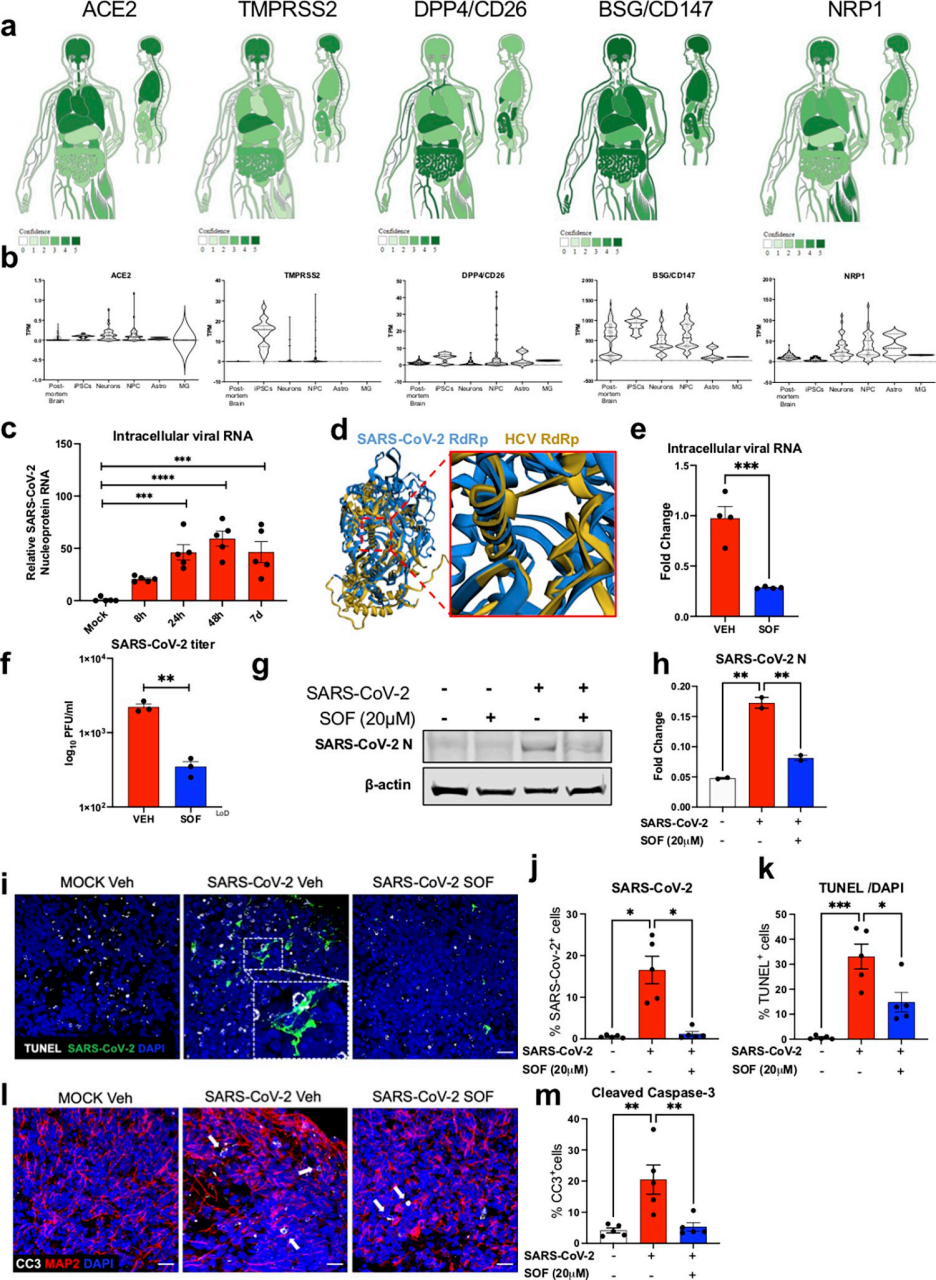
SARS-CoV-2 replicates in BCO and is inhibited by SOF treatment. **a**. Gene-tissue association based on transcriptomics and proteomics repositories generated using the Oxford database^45^. The scoring shows confidence of identification for the genes and proteins ACE2, DDP4/CD26, BSG/ CD147, TMPRSS2 and NRP1 in different human body tissues/organs both in coronal and sagittal planes; the darker the green tone is, the higher is the confidence score. **b**. Violin plots showing the frequency of distribution of normalized mRNA expression in adult postmortem brain (n = 172), induced pluripotent stem cells (iPSC, n = 18), neurons (n = 59), neural progenitor cells (NPC, n = 72), astrocytes (Astro, n = 3), and microglia (MG, n = 2) obtained from public databases^45–47^. All data points are represented as individual points inside each violin plot. **c.** Quantification of SARS-CoV-2 intracellular mRNA by qPCR of BCO infected at MOI 2.5. RNA was collected at 8, 24, 48 hours and 7 days. Bars represent mean. Error bars represent standard error mean (SEM) ***p<0.001, ****p<0.0001, n = 5 biological replicates (two pooled organoids per replicate, measured in triplicates), significance was assessed using one-way analysis of variance (ANOVA) with Dunnett’s post-hoc test. **d.** Structural superposition of SARS-CoV-2 RdRp (colored blue) and HCV RdRp (colored yellow) showing structural overlap within the polymerase active site. Both structures are statistically similar (p = 8.51e-07) calculated from raw FATCAT score^73^. A total of 389 equivalent positions with a root-mean-square deviation (RMSD) of 3.10Å and 2 twists were found between these two structures. **e.** Quantification of SARS-CoV-2 intracellular mRNA by qPCR of BCO infected at MOI 2.5 and treated with vehicle (Veh) or 20μM Sofosbuvir (SOF). RNA was collected 7 days post-infection. Bars represent mean. Error bars represent SEM ***p<0.001, n = 4 biological replicates (two pooled organoids per replicate). Significance was assessed using unpaired t-test. **f**. SARS-CoV-2 titer was determined by plaque assay. Supernatants from SARS-CoV-2 infected BCO at MOI 2.5 treated with vehicle (Veh) or 20μM Sofosbuvir (SOF), were collected at 16 h post-infection and subjected to plaque assays using Vero E6 cells. Plaques were quantified and recorded as log_10_PFU/ml. Bars represent mean. Error bars represent SEM **p<0.01, n = 3 biological replicates. Significance was assessed using unpaired t-test. LoD = limit of detection. **g.** Western-blot of SARS-CoV-2 Nucleocapsid (N) infected BCO at MOI 2.5 treated with vehicle (Veh) or 20μM Sofosbuvir (SOF) 7 days post-infection. Beta actin was used as a loading control. **h.** Western-blot analyses of SARS-CoV-2 Nucleocapsid (N) represented in g. Bars represent mean. Error bars represent SEM **p<0.01, n = 12 biological replicates (pooled into two protein lysates), Significance was assessed using one-way ANOVA and Tukey’s multiple comparisons test. **i.** Immunolabelling of TUNEL (white), SARS-CoV-2 nucleoprotein (N) (green) in mock, infected, and SOF-treated organoid sections. Scale bar, 20 μm. **j,k.** Quantification of the number of SARS-CoV-2^+^ and TUNEL^+^ cells with vehicle (Veh) or treatment with 20μM SOF respectively. n>10 biological replicates per condition (calculated in % compared to overall number of cells). **l.** Immunolabelling of CC3 (white) in mock, infected, and SOF treated organoid sections. Scale bar, 20 μm. **m.** Quantification of the number of CC3+ cells upon vehicle (Veh) or treatment with 20μM SOF, n = 5 biological replicates per condition. (calculated in % compared to overall number of cells). Error bars represent SEM *p<0.05, **p<0.01, Significance was assessed using one-way ANOVA and Tukey’s multiple comparisons test. Two different batches of BCOs from two different iPSC lines (WT83 and CVB) were used, and 5 organoids were analyzed per condition. Quantifications were done manually with 6 ROIs per sample. The raw data for the panels on this figure is located in S1 Data file.

We next focused on iPSC-derived and human primary CNS cells (Figs 1B, S1D and S1E). Expression of ACE2 was detected in iPSCs, neurons, neural progenitor cells (NPC), and astrocytes, but not in microglial cells (MG) (Fig 1B). TMPRSS2 was expressed in iPSCs and to a lesser extend in neurons and NPCs, but was not detected in adult postmortem brains tissue, iPSC-derived astrocytes, or MG (Fig 1B). Consistent with the previous database, BSG/CD147 was highly expressed in all samples, except for MG, which had the lowest level of expression. NRP1 or Neuropilin-1 was highly expressed in neurons, NPCs, and astrocytes but at a lower level in MG, iPSCs, and adult postmortem brains (Fig 1B). Finally, DPP4/CD26 was detected in all samples at similar low expression levels (Fig 1B). Altogether, the analyses of gene and protein expression levels of these viral entry factors suggest that the human brain might be susceptible to infections with SARS-CoV-2.

Based on cellular receptors expression data, we next tested whether SARS-CoV-2 could infect the developing human brain by generating eight-week-old human brain cortical organoids (BCO) from dermal fibroblasts from healthy donors. To evaluate if BCOs are susceptible to SARS-CoV-2, organoids were infected with the virus (isolate USA-WA1/2020) at a multiplicity of infection (MOI) of 2.5, a viral concentration similar to that used by several other reports using brain organoids [22–26]. We assessed viral replication by measuring intracellular viral RNA quantity over time. Quantification of intracellular SARS-CoV-2 RNA by qRT-PCR in infected BCOs revealed an increase in viral mRNA overtime that peaked at 48 hours post-infection and then declined over the course of infection (Fig 1C), suggesting the ability of BCOs to support the replication of SARS-CoV-2, which at this stage, are mainly composed of approximately 45% NPC, 41% neurons, and 14% astrocytes [37] (S2A and S2B Fig).

We next conducted RNA-seq analyses to evaluate changes in gene expression after virus challenge. Upon infection with SARS-CoV-2, we noted 477 differentially expressed genes at a 1.25-fold change (p value < 0.05) (S2C–S2E Fig and S1 Table). These factors were found to be enriched in pathways that have been previously associated with viral infection (p value < 0.05), including antigen presentation, viral entry via endocytic pathways, negative neuronal projection development, oxidative stress, and the complement pathway (S2D and S2E Fig and S2 Table).

Given the impact of SARS-CoV-2 on organoids, we next focused on how to alleviate its impact by testing FDA-approved antiviral drugs to possibly repurpose for SARS-CoV-2 infections. Despite vaccination efforts, there remains an urgent need to treat the increasing number of virus variants and, thus, COVID-19 infected patients. To this end, we tested Sofosbuvir (SOF, Sovaldi, Gilead Sciences) as an antiviral candidate. SOF is an FDA-approved anti-hepatitis C (HCV) treatment that blocks HCV replication by inhibiting its RNA-dependent RNA polymerase (RdRp) [48–50]. SOF can also suppress other viral families of single-stranded, positive-sense RNA viruses, including coronaviridae [51,52]. Previous reports suggested that SOF may penetrate the brain sufficiently to prevent any long-term, CNS-related sequelae [52,53]. The SARS-CoV-2 RdRp shares high sequence and structural homology with HCV [54], and SOF-binding residues are conserved amongst several coronaviruses, including SARS-CoV-2, SARS, and Middle East Respiratory Syndrome (MERS) [55], suggesting it could also inhibit SARS-CoV-2 replication [56]. Importantly, the structural superposition of SARS-CoV-2 RdRp (nsp12 domain) and HCV RdRp (non-structural protein 5B or NS5B domain) showed statistically significant similarity in structural overlap within the polymerase active site (Fig 1D). Therefore, we hypothesized that SOF could inhibit SARS-CoV-2 replication. We first determined a range of SOF dosages for BCO treatment based on our previous studies, where SOF prevented the vertical transmission of the Zika virus from pregnant dams to pups and protected the CNS of the newborns [52,57]. We then treated BCOs with incremental doses of SOF and found that SOF was able to reduce intracellular SARS-CoV-2 RNA levels in a dose-response manner (S2F and S2G Fig). Since treatment with 20 μM resulted in the highest inhibition of SARS-CoV-2 replication without inducing any cell death (S2G and S2H Fig), this dose was chosen for subsequent experiments.

To further confirm whether BCOs support SARS-CoV-2 growth and whether this is inhibited by SOF treatment, we measured the intracellular viral RNA and the number of infectious viruses present in the supernatants of SARS-CoV-2-infected BCOs at 16 hours post-infection. Notably, the amount of retrieved infectious viruses was significantly reduced upon SOF treatment (a 5-fold decrease in the supernatant and by 75% for the intracellular viral RNA), further supporting both the ability of BCOs to allow productive replication of SARS-CoV-2 and SOF as an inhibitor of viral growth (Fig 1E and 1F). Subsequent immunoblotting and immunostaining experiments on SARS-CoV-2-infected BCOs 7 days post-infection revealed that SOF treatment also reduced SARS-CoV-2 nucleoprotein (N) protein levels (Fig 1D–1J). Notably, the immunostaining revealed a significant increase in cell death measured by both cleaved caspase 3 (CC3) by 20% and TUNEL immunostainings by 30%, accompanied by a 15% increase in the amount of SARS-CoV-2^+^ cells in virus-infected BCO when compared to non-infected controls (Fig 1J, 1K, and 1M). Treatment with SOF significantly decreased both SARS-CoV-2 viral protein levels and viral-induced cell death (Figs 1E, 1J, 1K, 1M, and S2G–S2I).

We next examined the targeted cell types and cell type-specific susceptibility to SARS-CoV-2 infection. We analyzed SARS-CoV-2 infection and colocalization within each cell population. Both Nestin^+^ NPCs and MAP2^+^ neurons showed similarly significant increases in SARS-CoV-2 NP staining (Figs 2A–2F, S3A, and S3B). We also detected limited viral staining in GFAP^+^ astrocytes in our experimental conditions (Figs 2G–2I and S3D). The increase in viral N protein presence in BCO was accompanied by increased cell death. We detected similar proportions of increase in cell death both in NPCs (identified both with Nestin and SOX2 antibodies) and neurons (Figs 2A–2F and S3A–S3C), while GFAP^+^ astrocyte did not show any statistically significant differences compared to mock conditions (Figs 2G–2I and S3A–S3C and S1–S3 Videos). Interestingly, we noted that not all SARS-CoV-2 N^+^ cells were TUNEL^+^ or vice versa, suggesting that SARS-CoV-2 could have an indirect, non-cell autonomous bystander effect, ultimately leading to the death of non-infected cells.

**Fig 2:**
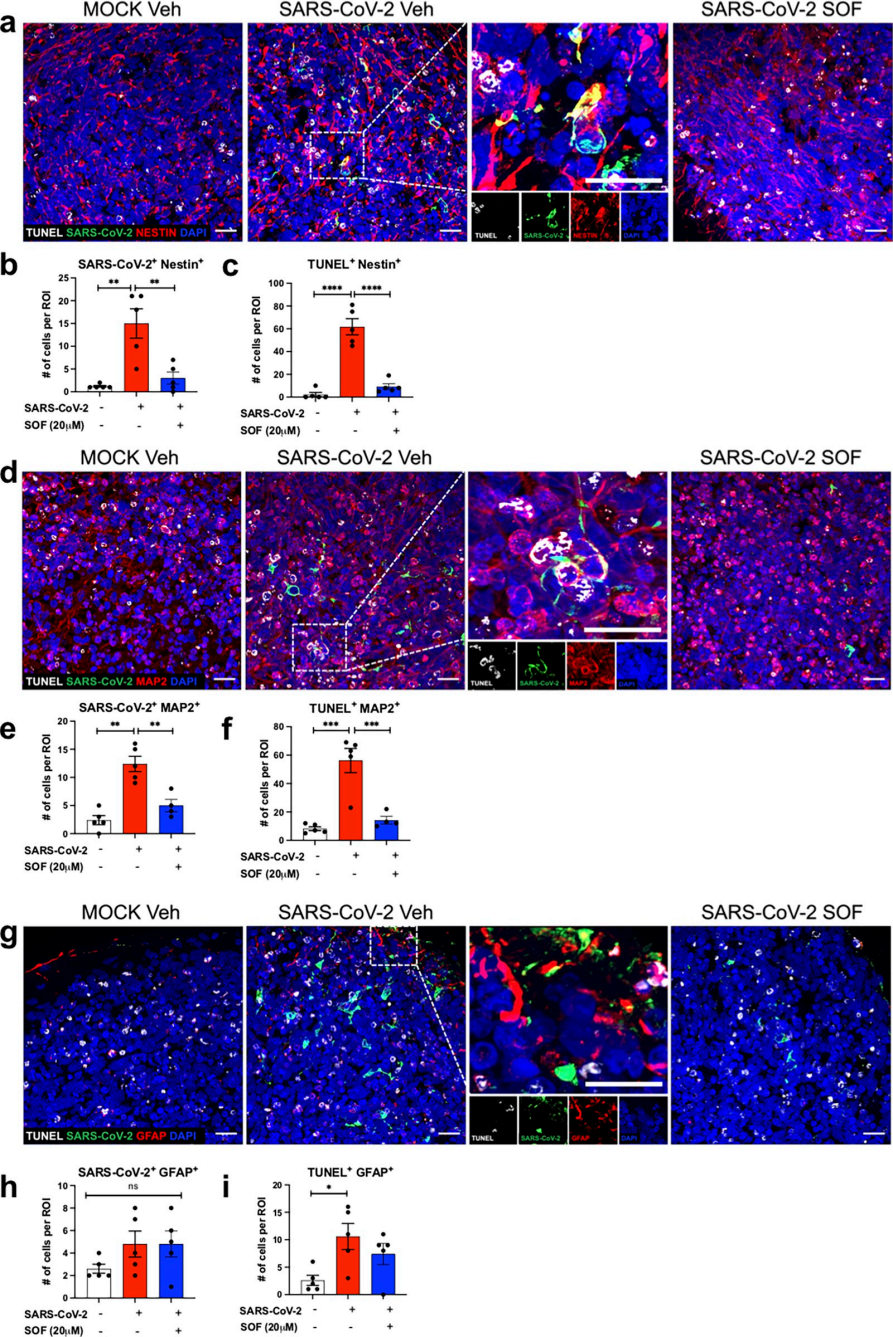
SARS-CoV-2 impact on different brain cell types. **a-d-g**. Immunolabeling of organoid sections stained for TUNEL (white), SARS-CoV-2 N protein (green), Nestin (red), GFAP (red), MAP2 (red), respectively by confocal microscopy. The insets show merged images and depict colocalization of TUNEL or SARS-CoV-2 N protein within each cell type; Nestin^+^ NPC, GFAP^+^ astrocytes or MAP2^+^ neurons. Images below each inset show split channels. Scale bar, 20 μm n = 5 biological replicates per condition. **b,c,e,f,h,i.** Quantification of the number of cells that are Nestin^+^, MAP2^+^ or GFAP^+^ cells (red) in BCO colocalizing with SARS-CoV-2 N-positive cells (green) or TUNEL (white), MOI 2.5, n = 5 biological replicates per condition. Bars represent mean, error bars represent SEM **p<0.01, ****p<0.0001 significance was assessed using one-way ANOVA multiple comparisons test. The number corresponding to TUNEL^+^ and SARS-CoV-2^+^ cells within different NPC (Nestin^+^), astrocyte (GFAP^+^) and neuronal (MAP2^+^) populations were counted per region of interest (ROI). Two different batches of BCOs from two different iPSC lines (WT83 and CVB) were used, and 5 organoids were analyzed per condition. Quantifications were done manually with 6 ROIs per sample, ROIs were chosen blindly. Bars represent mean. Error bars represent SEM, n = 3 biological replicates (four pooled organoids per replicate). Significance was assessed using one-way ANOVA with Dunnett’s post-hoc test. The BCOs were fixed and analyzed 7 days post-infection. The raw data for the panels in this figure is located on S1 Data file.

Given that SARS-CoV-2 could infect MAP2^+^ post-mitotic neurons, we next analyzed the composition of excitatory neurons to validate their cortical identity and to investigate their susceptibility to SARS-CoV-2 infections (S4 Fig). The BCO contained both lower (CTIP2^+^), intermediate progenitors (TBR2^+^) and upper layer neurons (SATB2^+^ and CUX1^+^) which were all susceptible to SARS-CoV-2 infections with a preferential infection of upper layer neurons as they co-stained with the cell death marker TUNEL (S4 Fig).

As neurons from different cortical layers showed increased cell death following SARS-CoV-2 infections, we next evaluated whether the infection could also affect glutamatergic synapse number/synapse formation. To assess the impact of SARS-CoV-2 on synaptic integrity, we quantified the number of excitatory synapses in neurons using vGLUT1, Synapsin 1 and PSD95 antibodies (Fig 3A–3G). The pre-synaptic proteins vGLUT1 and Synapsin 1 were decreased by 70% in MAP2^+^ cells (60% in overall cells) and by 60%, respectively, upon infection with SARS-CoV-2 compared to mock conditions, which were rescued upon treatment with SOF post-infection (Figs 3A–3F, S5A, S5B, S6B, and S6C). However, the post-synaptic protein PSD95 did not differ significantly upon viral infection (Figs 3A–3F, S5A, S5B, S6B, and S6C). Co-localized pre- and post-synaptic markers (Synapsin 1 and PSD95) also showed a significant reduction in infected BCO, which was also rescued with SOF treatment post-infection (Figs 3G and S6C). To understand whether SARS-CoV-2 had a direct impact on neurons, we co-stained MAP2^+^ neurons with SARS-CoV-2 antibodies and TUNEL (S5A Fig). We found little to no MAP2^+^/SARS-CoV-2^+^/TUNEL^+^ cells, suggesting that either dying neurons lose the SARS-CoV-2 stain or that the effect of SARS-CoV-2 on neurons could be through a non-cell autonomous mechanism (S5A Fig). We have also attempted to investigate whether the MAP2^+^/TUNEL^+^ were the ones losing their pre-synaptic connections through a co-stain with SYN1^+^. However, we could not conclude for certainty as tracing dying neurons is challenging in a tridimensional setting (S5B Fig).

**Fig 3:**
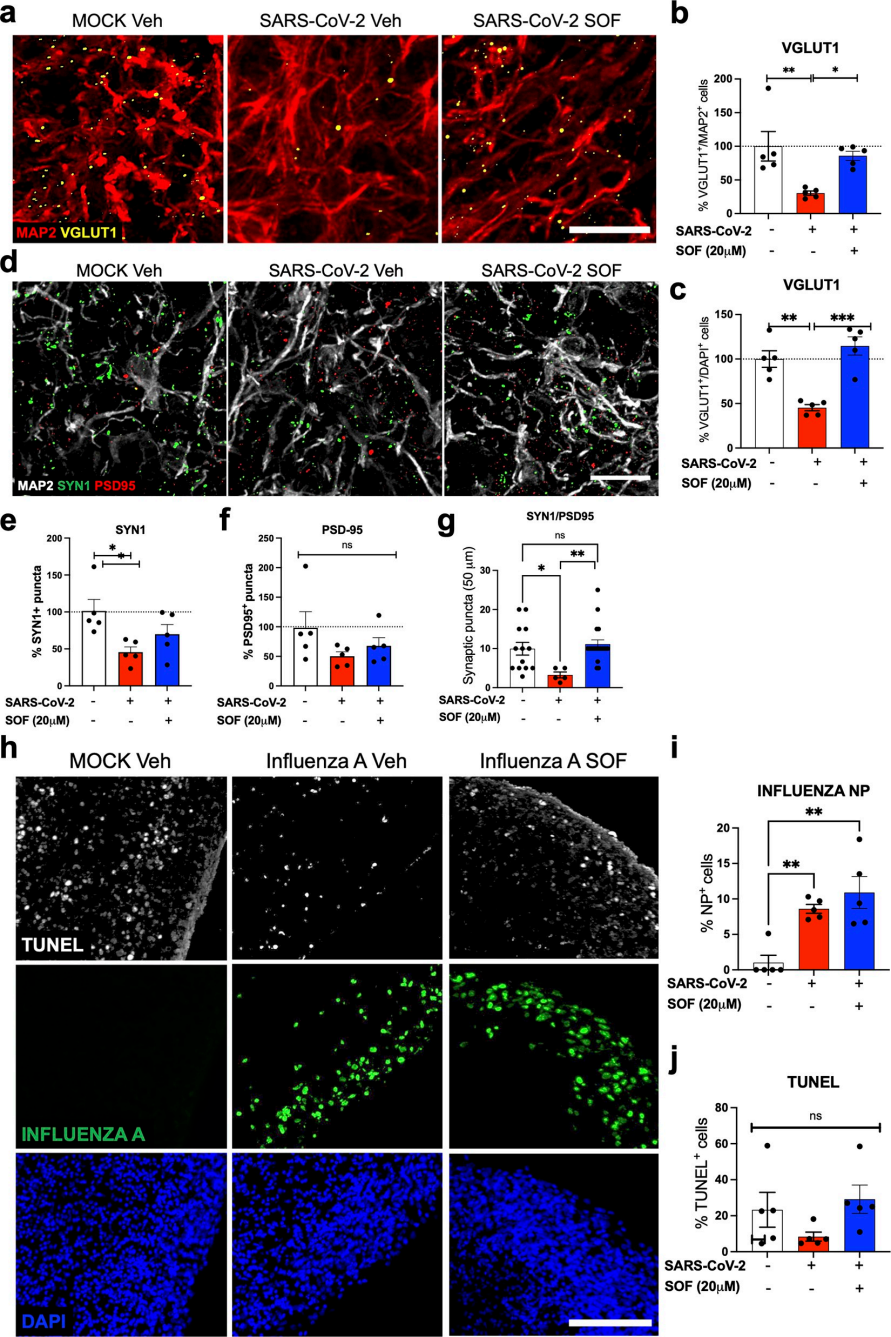
SARS-CoV-2 decreases excitatory synapses in BCO. **a,b,c.** Immunolabeling and quantification of vGLUT1 positive cells (yellow) within MAP2^+^ neurons (red) or compared to overall cells in BCO infected at MOI 2.5 and treated with vehicle (Veh) or 20μM Sofosbuvir (SOF) n = 5 biological replicates per condition, calculated in % compared to mock conditions. Bars represent mean. Error bars represent SEM. *p<0.05, **p<0.01. Significance was assessed by one-way ANOVA and Tukey’s multiple comparisons test. Scale bar, 20 μm. **d, e, f.** Immunolabeling and quantification of Synapsin 1- (SYN1) and PSD95-positive cells (green and red respectively) within MAP2^+^ neurons (white) in BCO infected at MOI 2.5 and treated with vehicle (Veh) or 20μM Sofosbuvir (SOF), n = 5 biological replicates per condition, calculated in % compared to mock conditions. Scale bar, 20 μm. **g**, Quantification of Synapsin 1- (SYN1) and PSD95-positive co-localized puncta in BCO infected at MOI 10 and treated with vehicle (Veh) or 20μM Sofosbuvir (SOF). Two different batches of BCOs from two different iPSC lines (WT83 and CVB) were used, and 3 ROI from each of the 5 organoids were analyzed per condition. Data is normalized to mock vehicle conditions. Bars represent mean. Error bars represent SEM. *p<0.05, **p<0.01. Significance was assessed by one-way ANOVA and Tukey’s multiple comparisons test. **h.** Immunolabeling of TUNEL (white) and Influenza A virus nucleoprotein (NP, green) in BCO infected at MOI 2.5 and treated with vehicle (Veh) or Sofosbuvir (SOF). Scale bar, 100 μm. **i, j**. Quantification of Influenza A virus NP protein and TUNEL positive cells. Two different batches of BCOs from two different iPSC lines (WT83 and CVB) were used, and 5 organoids were analyzed per condition. Quantifications were done manually with 6 ROIs per sample. Data is normalized to mock vehicle conditions (calculated in % compared to mock conditions). Error bars represent SEM **p<0.01, ***p<0.001, statistical significance was assessed with one-way ANOVA multiple comparisons test. The BCOs were fixed and analyzed 7 days post-infection. The raw data for the panels on this figure is located in S1 Data file.

We also studied the impact of the infection on overall astrocytic populations (S5C–S5D Fig). Infection with SARS-CoV-2 did not change the proportion of GFAP^+^, Vimentin^+^ or Aquaporin-4^+^ cells, suggesting that the virus is not actively killing these cells (S5C and S5D). Moreover, although SOF did not induce cell death in mock cells, we confirmed that the treatment with SOF of mock-infected BCO did not induce any changes in the number of PSD95^+^ or SYN1^+^ puncta (S6A Fig).

As a control for our studies, we assessed the impact of another respiratory virus, Influenza A virus, on BCO using the same experimental design (Figs 3H–3J, and S6D). Interestingly, while we noted an accumulation of viral proteins in infected BCO upon infection as measured by Influenza A virus NP immunostaining (Fig 3H–3J), this was not accompanied by an increase in cell death as measured by TUNEL staining (Fig j). Also, treatment with SOF on BCOs infected with Influenza A failed to decrease the accumulation of viral proteins (Fig 3H and 3I), suggesting a treatment specificity towards SARS-CoV-2. We aligned the polymerase structures of SARS-CoV-2 (RdRp) and Influenza A virus Polymerase Basic protein 2 (PB2) and found no significant overlap, suggesting that SOF might not be able to bind to the polymerase site (S6E Fig). The RdRp domain of HCV contains an active site with a GDD motif where SOF docks to inhibit its replication [52,58,59]. The sequence alignment of the polymerase sequences of SARS-CoV-2 (RdRp), HCV (NS5B), and Influenza A PB2 showed that the polymerase domain of SARS-CoV-2 had more conserved residues with the polymerase domain of HCV compared to the Influenza A virus (S6E–S6G Fig). Importantly, our sequence alignment showed that the GDD motif was more conserved in SARS-CoV-2 compared to the Influenza A virus, possibly explaining the failure of SOF to rescue the accumulation of viral proteins upon infection with Influenza A virus (S6E–S6G Fig).

The immediate and long-term lasting neurological and neuropsychiatric sequelae of COVID-19 are currently surfacing, but it might still take several years to document the survivors’ cognitive and mental health burden of recovered COVID-19 cases [60]. Several other publications using human brain organoids have also found that SARS-CoV-2 impacted neural cells with some disparities [22–24,26,61,62]. These disparities could be due to the differences in different brain organoid protocols, analyses timepoint post-infection, and MOIs used, which should be considered when comparing different studies [26]. However, even with these experimental differences, we have found the same molecular pathways that were dysregulated in the SARS-CoV-2 infected BCOs, including oxidative stress, antigen presentation, viral entry via endocytic pathways, negative neuron projection development, and the complement pathway [23,61,62], supporting previous findings on the impact of SARS-CoV-2 on a developing human brain. Our findings show that SARS-CoV-2 can infect different cell types in human BCO at a similar MOI that was previously used by other groups [22–24,61,63]. Moreover, we show that SARS-CoV-2 rapidly decreased the number of excitatory synapses in neurons within seven days post-infection, revealing a potential novel mechanism for the associated neurological symptoms with COVID-19. However, one caveat of our study is that we have not investigated whether synaptic transmission or physiology is altered in SARS-CoV-2 infected BCOs due to safety issues (such work must be done inside a BSL3 facility). Although the decrease in the amount of pre-synaptic proteins vGLUT1 and Synapsin 1 might be a result of neuronal death, we cannot exclude the possibility of a non-cell autonomous toxic effect coming from other non-neuronal cells such as astrocytes [23,63]. Our data add to the experimental evidence that the developing human brain is susceptible to SARS-CoV-2 infections, potentially leading to long-term impairments in neuronal function, perhaps through alterations in ApoE4 and Tau proteins as suggested by others [22,63].

While the current predominant theory for neurological symptoms of COVID19 is through vascular abnormalities, in our study, we mainly focused on the direct impact of SARS-CoV-2 on BCO *in vitro*. So far, there is limited *in vivo* evidence as to whether SARS-CoV-2 affects the brain or if/how SARS-CoV-2 gains access to the human brain. Two reports, however, suggested that choroid plexus cells are susceptible to SARS-CoV-2 infections [23,24], which could be one way how the virus gains access to the brain parenchyma. Other non-exclusive ways that SARS-CoV-2 could access the brain include through infection of vascular endothelium and leukocyte transmigration across the blood-brain barrier [29].

Supporting our data, Song and colleagues have recently used multiple experimental models to evaluate the impact of SARS-CoV-2 on human and mouse central nervous system [61]. By using human brain organoids, a genetically modified mouse model and autopsies from patients who died of COVID-19. Song et al., provided compelling evidence that SARS-CoV-2 does indeed have a neuroinvasive capacity [61]. The authors showed that SARS-CoV-2 could efficiently replicate in the mouse brain, leading to CNS-specific lethality within days after viral infection [61], warranting against worse outcomes for COVID-19 patients with neurological symptoms in the future.

Another recent publication studying the impact of SARS-CoV-2 on *postmortem* tissues of COVID-19 patients has found dramatic inflammation and T-cell infiltration in the brain [27]. Moreover, the authors also noted deep synaptic alterations and transcriptional changes similar to those with chronic neurological disorders [27]. These alarming findings not only support and validate our findings but also warrant the need for urgent anti-viral treatments in addition to vaccinations to prevent further long-term CNS damage.

Amid the recently detected SARS-CoV-2 variants with a higher transmissibility rate according to the European Centre for Disease Prevention and Control, the rapidly increasing number of infected patients and the clinical outcome regarding the neurological symptoms of infected patients, efficient drug treatment for COVID-19 is urgently in need. We previously used human brain organoids to repurpose drugs for several neurological conditions [52,64–66]. Here, we found that SOF can inhibit SARS-CoV-2 replication in human brain cells post-infection and rescue the observed neurological impairments. Supporting our findings, SOF has been pointed as a potential treatment against COVID-19 based on *in silico* modeling [48,56,67]. Thus, treatment with SOF could also arrest or prevent the development of neurological symptoms in COVID-19 patients. Because SOF did not show safety concerns in pregnant women [68], it could also be an option to block a possible vertical transmission from SARS-CoV-2-infected pregnant women for whom prevention is no longer an option. Although further clinical studies are needed, we provide initial evidence that SOF could be an immediate candidate to pharmacologically treat COVID-19 and related neurological manifestations.

## Materials and Methods

### Transcriptomic analyses and gene expression profile obtained from publicly available databases

To obtain the expression profile of the human genes *ACE2*, *DDP4*, *BSG and TMPRSS2* and *NRP1* corresponding coded proteins, the online resource TISSUES 2.0 was used to show the confidence of the expression for the investigated genes [45], with 5 stars corresponding to the highest confidence. To obtain the gene and protein expression profiles, we used GTEx (GTEx Consortium, 2013) [46] and Human Protein Atlas [47] to show normalized expression values [45].

For each of the three transcriptomics datasets (HPA, GTEx and FANTOM5), the average TPM value of all individual samples for each human tissue or human cell type was used to estimate the gene expression level. To combine the datasets into consensus transcript expression levels, a pipeline was set up to normalize the data for all samples. In brief, all TPM values per sample were scaled to a sum of 1 million TPM (denoted pTPM) to compensate for the non-coding transcripts that had been previously removed. Next, all TPM values of all the samples within each data source (HPA human tissues, HPA blood cells, GTEx, and FANTOM5 respectively) were TMM (trimmed mean of M values) normalized, followed by Pareto scaling of each gene within each data source. Tissue data from the three transcriptomics datasets were subsequently integrated using batch correction through the removeBatchEffect function of R package Limma, using the data source as a batch parameter. The blood RNA-seq dataset was not limma-adjusted. The resulting transcript expression values, denoted Normalized eXpression (NX), were calculated for each gene in every sample.

In the Human Protein Atlas, the NX value for every gene and tissue were calculated and visualized on the gene summary page together with the pTPM values for the individual samples. **Consensus** transcript expression levels for each gene were summarized in 74 human tissues based on transcriptomics data from three sources: HPA, GTEx and FANTOM5. The consensus normalized expression (NX) value for each gene and organ/tissue represents the maximum NX value in the three data sources. For tissues with multiple sub-tissues (brain regions, blood cells, lymphoid tissues and intestine) the maximum of all sub-tissues is used for the tissue type. The total number of tissue types in the human tissue consensus set is 37 and the total number of human blood cell types is 18. The following BioProject codes and corresponding cell type and/or tissue samples were included within our research: BioProject PRJNA314463, PRJNA527289, PRJNA343829, PRJNA231202, PRJNA398545, PRJNA143369, PRJNA316853, PRJNA222268, PRJNA254971, PRJNA302685: human *postmortem* brain tissue samples; BioProject PRJNA261255: human neuronal progenitor cells; BioProject PRJNA253946: human neuron cells; BioProject PRJNA224073: human iPSC and ESC cells; BioProject PRJNA280163: human iPSC, neurons and neuronal progenitor cells; BioProject PRJNA419983: human iPSC, neurons, neuronal progenitor cells and post mortem brain; BioProject PRJNA358689: human neurons; BioProject PRJNA291180: human neurons and neuronal progenitor cells; BioProject PRJNA248182: human neurons; BioProject PRJNA417295: human neurons and neuronal progenitor cells; BioProject PRJNA350562: human fetal microglia.

### Sequence alignments between SARS-CoV-2, HCV and Influenza A

Sequence alignments were performed using T-COFFEE [69] (v11.00.d625267, Build 507), which employs several established alignment algorithms to generate a consensus alignment of queried sequences. For alignment between SARS-CoV-2 RdRP (YP_009725307.1) and HCV NS5B (YP_009709870.1) and Influenza A PB2 (NP_040987.1), all available pairwise alignment algorithms within the M-COFFEE suite (Mlalign_id, Mclustalw, Mpoa, Mprobcons, Mfast, Mproba, Mmafft, Mdialigntx, Mslow, Mpcma, Mmuscle) were employed. The consensus alignment was then trimmed to the region of interest with Jalview v2.11 and shaded using Boxshade v3.21. Residues involved in SOF binding or catalytic activity [55] are highlighted in yellow to signify mismatches orange to signify partial matches and red to signify matches.

Pairwise structural alignments between SARS-CoV-2 RdRp bound to Remdesivir and RNA template (PDB ID: 7BV2 [70] chain A) and Hepatitis C virus RdRp bound to Sofosbuvir and RNA template (PDB ID: 4WTG [71] chain A) and Influenza A RdRp bound to RNA template (PDB ID: 4WSB [72] chain B) were performed using FATCAT 2.0 [73], which optimizes alignment and minimizes the number of rigid-body movements (twists) around pivot points (hinges) of flexible protein structures. Structural superpositions were visualized using JMol v14.

### Viruses

SARS-CoV-2 USA-WA1/2020 strain, isolated from an oropharyngeal swab from a patient with a respiratory illness who developed clinical disease (COVID-19) in January 2020 in Washington, USA, was obtained from BEI Resources (NR-52281). The virus was propagated in Vero E6 cells (ATCC CRL-1586TM) transfected with exogenous human ACE2 and TMPRSS2. Virus titers were determined by plaque assay performed on Vero E6 cells. All experiments involving live SARS-CoV-2 followed the approved standard operating procedures of the Biosafety Level 3 facility at the Sanford Burnham Prebys Medical Discovery Institute. A/WSN/1933 (H1N1) influenza A virus was propagated in MDCK cells (ATCC CCL-34). Virus titer was determined by plaque assay on MDCK cells using agar overlay medium.

### Cell lines

Vero E6 and MDCK cells were maintained in Dulbecco’s modified eagle medium (DMEM, Gibco) supplemented with 10% heat-inactivated fetal bovine serum (FBS, Gibco), 50 U/mL penicillin, 50 μg/mL streptomycin, 1 mM sodium pyruvate (Gibco), 10 mM HEPES (Gibco), and 1X non-essential amino acids solution (Gibco).

Donated healthy fibroblasts were obtained via skin biopsies from patients after informed consent was appropriately given under protocols approved by the University of California, San Diego Institutional Review Board (#141223ZF). All experiments were approved and performed under the Institutional Review Boards (IRB) and Embryonic Stem Cell Research Oversight (ESCRO) guidelines and regulations.

### Cell culture

Two iPSC lines from healthy donors (WT83 and CVB) were cultured and manually passaged onto Matrigel-coated (Corning) and fed daily with mTESR1 (StemCell Technologies) [74–76].

BCO generated from the two different healthy control iPSC lines were differentiated as previously described [37]. Briefly, iPSCs were dissociated using a 1:1 Dulbecco’s phosphate-buffered saline (DPBS, ThermoFisher) and StemPro Accutase (ThermoFisher) solution. Cells were centrifuged and resuspended in mTeSR1 supplemented with 10 μM SB431542 (SB; Stemgent) and 1 μM Dorsomorphin (R&D Systems). 4x10^6^ cells were transferred to one well of a 6-well plate and kept in suspension under rotation (95 rpm) for 24 hours with 5 μM of ROCK inhibitor (Y-27632; Calbiochem). Forty-eight hours later, media was substituted by the neural induction media consisting of DMEM/F12 (Life Technologies), 1% Glutamax (Life Technologies), 1% N2 Neuroplex (Gemini Bio), 1% non-essential amino acids (NEAA, Gibco), 1% Pen-Strep (PS; ThermoFisher), 10μM SB431542 and 1μM of Dorsomorphin. The media was changed every other day for seven days. Media was substituted for neural proliferation media consisting of Neurobasal media (Life Technologies), 2% Gem21 Neuroplex, 1% non-essential amino acids, 1% Glutamax, and 20 ng/mL basic fibroblast growth factor (bFGF; Life Technologies). The media was changed daily for 7 days, followed by another seven days of neural proliferation media supplemented with 20 ng/mL of epidermal growth factor (EGF, Peprotech). Neuronal Maturation step was achieved by changing the media to Neurobasal with GlutaMAX, 1% Gem21 NeuroPlex (Gemini Bio), 1% NEAA and 1% PS; supplemented with 10 ng/mL of BDNF, 10 ng/mL of GDNF, 10 ng/mL of NT-3 (PeproTech), 200 mM L-ascorbic acid and 1 mM dibutyryl-cAMP (Sigma-Aldrich). The media was replaced every other day for seven more days. We executed cortical organoid experiments using maintenance media: Neurobasal with GlutaMAX, 1% Gem21 NeuroPlex (Gemini Bio), 1% NEAA, and 1% PS.

All the cell lines tested negative for mycoplasma contamination. All cell lines used have been authenticated [74–76].

### *In vitro* infection

BCO were infected with SARS-CoV-2 or Influenza A virus H1N1 on day 52 of differentiation using maintenance media. BCO were infected with 750,000 PFU (corresponding to a MOI of 2.5 considering an average of 300,000 cells per organoid) for 1 hour at room temperature. Viral inoculum was removed, and cells were washed twice before fresh media was added atop including DMSO (vehicle) or indicated doses of Sofosbuvir (SOF; Acme Bioscience AB3793). Drugs were readded every second day for indicated times. BCOs supernatants were subjected to plaque assays (see below), and BCOs were subjected to western blot analysis (see below), viral isolation and qRT-PCR analysis (see below), or immunostaining. For immunostaining, BCOs were fixed for 48 hours with a 4% paraformaldehyde (PFA) solution, and further embedded in 30% sucrose for 24 hours. Tissue was placed in a mold for cryosectioning and covered with a layer of optimal cutting temperature (CRYO-OCT; VWR) compound to prevent freeze-drying and store the rest of the sample at −80°C. Samples were sliced in 20μm cryosections using (Leica CM3050) and prepared for immunostaining (see below for further details).

### Western blot

Total protein (20μg) was separated using a Bolt 4–12% Bis-Tris Plus Gel (Life Technologies, Carlsbad, CA, USA) and transferred to a nitrocellulose membrane using an iBlot2 dry blotting system (Thermo Scientific, Waltham, MA, USA). Membranes were blocked for two hours (Rockland Immunochemicals, VWR International, Arlington Heights, IL, USA), and primary antibodies (Anti-SARS-CoV-2 N antibody (rabbit, 1:10,000, gift from Kwok-Yung Yuen, University of Hong Kong), Anti-β-actin, (mouse, Abcam ab8226, 1:10,000) in blocking buffer) incubated, shaking, overnight at 4°C. Membranes were washed three times, five minutes each, in PBST, after which the secondary antibodies (IRDye 680RD and IRDye 800CW, 1:5,000 in blocking buffer) incubated for one hour at room temperature. Following three more membrane washes in PBST, the proteins were detected by an Odyssey CLx infrared imaging system (LiCOR Biosciences, Lincoln, NE, USA); signal intensity was corrected to the quantification of β-actin.

### Immunofluorescence

BCO were fixed with 4% paraformaldehyde for 48 hours at 4°C. Next, samples were permeabilized in 1xPBS (Corning) containing 0.1% (v/v) Triton X-100 for 10 minutes. Fixed cells were next incubated with blocking solution for 1 hour [3% Bovine Serum Albumin (BSA); (Gemini) in 1xPBS]. Primary antibodies were diluted with blocking solution and incubated with cells overnight at 4°C: Cleaved caspase-3 (rabbit, Cell Signaling #9661, 1:500), Anti-Nestin (mouse, Anti-Nestin antibody [10C2]; 1:1000), Anti-MAP2 antibody (Chicken, Abcam ab5392, 1:1000), Anti-vGLUT1 [317D4] (mouse, Synaptic Systems #135311, 1:500), Anti-Synapsin1, (rabbit, EMD-Millipore AB1543P, 1:1,500); Anti-PSD-95, (mouse Neuromab, 1:1,500); anti-CUX1 (rabbit, Sigma Aldrich, HPA003317, 1:500), anti-SATB2 (Mouse, Abcam, ab34735, 1:500), anti-TBR2 (Chicken, R&D, AF6166, 1:500), anti-Vimentin (Mouse, Abcam, ab8978, 1:500), anti-Aquaporin-4 (Rabbit, Abcam, ab46182, 1:500), Anti-GFAP (chicken, Abcam ab4674, 1:1000), anti-SOX2, (Rabbit, Cell Signaling, 2748S, 1:500), influenza A virus nucleoprotein (NP) HT-103 mouse monoclonal antibody (Mount Sinai, in-house antibody, 1:2,000). SARS-CoV-2 (2019-nCoV) Nucleoprotein (N) Antibody (rabbit Mab, Sino Biological #40143-R019, 1: 2000) was incubated diluted with blocking solution and incubated with cells for 20 minutes at room temperature. Cells were then washed twice with 1xPBS and incubated with the secondary antibody for 30 minutes at room temperature. Secondary antibodies (all conjugated to Alexa Fluor 488, 555 and 647) were purchased from Life Technologies and used at a 1:1000 dilution. After the 30 minutes incubation, samples were washed twice (1xPBS), incubated for 5 minutes with fluorescent nuclear DAPI stain (VWR; 1:10000), and mounted with Slow fade gold antifade reagent (Life Technologies). Samples were imaged using an Axio Observer Z1 Microscope with ApoTome (Zeiss). For TUNEL analysis, samples were fixed with 4% paraformaldehyde and permeabilized with 0.25% Triton X-100 for 15 minutes and then stained for TUNEL following manufacturer’s instructions (Click-iT TUNEL assay kit from Life Technologies). Cells were blocked with 3% BSA for 1 hour and then incubated in primary and secondaries, as shown above. Images were blindly collected using an Axio Observer Z1 Microscope with ApoTome (Zeiss) and analyzed with ImageJ software.

### Immunofluorescence Image Analyses

We quantified the number of fluorescent cells corresponding to each staining was measured automatically with Image J software by splitting the different channels. The data is represented as percentage of positive cells for a specific staining overall the total number of the cells (DAPI) in each region of interest (ROI) or as the number of cells per ROI. Two different batches of BCOs from two different iPSC lines were used, and 5 organoids were analyzed per condition. Quantifications was done manually with 6 ROIs per sample. The quantifications were performed blindly. To generate the videos, 10–12 Z-stack images were acquired per condition with Axio Observer Z1 Microscope with ApoTome (Zeiss) and 3D reconstructions were generated using Zeiss Zen Microscope Software and exported as.MOV.

### RNA extraction and viral qPCR

Total RNA was purified from infected cells using the NucleoSpin 96 RNA Kit (Takara) in accordance with the manufacturer’s instructions. The viral mRNA was quantified using TaqPath one-step RT-qPCR Master Mix (Thermofisher) using ActinB CTRL Mix (Thermofisher) as housekeeping gene, and the following primers and probe for qPCR measurements of viral genes, targeting SARS-CoV-2 Nucleoprotein:

N-Fwd: 5’-TTACAAACATTGGCCGCAAA-3’; N-Rev: 5’-GCGCGACATTCCGAAGAA-3’; N-Probe: 5’-FAM-ACAATTTGCCCCCAGCGCTTCAG-BHQ-3’. ΔΔCT was used to calculate the fold changes relative to the controls.

### RNA-seq sample preparation

RNA from BCO infected at MOI 2.5 and treated with vehicle (Veh) or 20μM Sofosbuvir (SOF) was extracted 7 days post-infection. In brief, three samples per condition, each consisting of two pooled BCOs, were processed following RNeasy Mini Kit (Qiagen #74104) instructions to extract total RNA. RNA was assessed for quality using an Agilent Tapestation 4200, and samples with an RNA Integrity Number (RIN) greater than 8.0 were used to generate RNA sequencing libraries using the TruSeq Stranded mRNA Sample Prep Kit with TruSeq Unique Dual Indexes (Illumina, San Diego, CA). Samples were processed following manufacturer’s instructions, modifying RNA shear time to five minutes. Resulting libraries were multiplexed and sequenced with 100 basepair (bp) paired end reads (PE100) to a depth of approximately 25 million reads per sample on an Illumina NovaSeq 6000. Samples were demultiplexed using bcl2fastq v2.20 Conversion Software (Illumina, San Diego, CA).

### RNA-seq data analysis

Data was analyzed by ROSALIND (https://rosalind.onramp.bio/), with a HyperScale architecture developed by OnRamp BioInformatics, Inc. (San Diego, CA). Reads were trimmed using cutadapt [77]. Quality scores were assessed using FastQC [78]. Reads were aligned to the Homo sapiens genome build hg19 using STAR [79]. Individual sample reads were quantified using HTseq [80] and normalized via Relative Log Expression (RLE) using DESeq2 R library [81]. Read Distribution percentages, violin plots, identity heatmaps, and sample MDS plots were generated as part of the QC step using RSeQC [82]. DEseq2 was also used to calculate fold changes and p-values and perform optional covariate correction. Clustering of genes for the final heatmap of differentially expressed genes was done using the PAM (Partitioning Around Medoids) method using the fpc R library [83]. Hypergeometric distribution was used to analyze the enrichment of pathways, gene ontology, domain structure, and other ontologies. The topGO R library [84], was used to determine local similarities and dependencies between GO terms in order to perform Elim pruning correction. Several database sources were referenced for enrichment analysis, including Interpro [85], NCBI [86], MSigDB [87,88], REACTOME [89], WikiPathways [90]. Enrichment was calculated relative to a set of background genes relevant for the experiment. Each sample represents a pool of 3 organoids.

The functional network analyses were generated through the use of Ingenuity Pathway Analysis (IPA) (QIAGEN Inc., https://www.qiagenbio- informatics.com/products/ingenuity-pathway-analysis) [91] and Metascape [92]. Terms with a p value < 0.01, a minimum count of 3, and a ratio between the observed and the expected by chance count of > 1.5, were collected and grouped into clusters based on their membership similarities. Here, p values are calculated based on the accumulative hypergeometric distribution. Sequencing information is summarized in S3 Table.

### Plaque assay

Supernatants from SARS-CoV-2 infected brain organoids were collected at 16 h post-infection and stored at -80°C until used. 600,000 Vero E6 cells were seeded overnight at 37°C / 5% CO2 in 12-well plates. Confluent Vero E6 cells were then washed once with 1xPBS and infected with 10-fold serial dilutions of the collected supernatants. Cells were incubated with the virus for 1 h at room temperature, followed by inoculum removal and addition of 1ml overlay media (2xMEM and 2.5% Avicel (FMC BioPolymer, RC-591 NF) at a 1:1 ratio). 2xMEM contains 100 ml 10x MEM (Gibco), 10 ml 100x penicillin-streptomycin (Fisher Scientific), 10 ml 100x L-Glutamine, 6 ml 35% BSA, 10 ml 10 mM 4-(2-hydroxyethyl)-1-piperazineethanesulfonic acid (HEPES, Gibco), 24 mL 5% NaHCO3 (Gibco) and 340 ml water. Plates were incubated 3 days at 37°C, 5%CO2, and then fixed and stained using 0.1% Crystal Violet and 5% PFA (Boston BioProducts) overnight at 4°C. Plaques were quantified and recorded as PFU/ml.

### Viability assay

BCO were treated with increasing doses of SOF or DMSO (vehicle control). Drugs were reapplied every second day for a total duration of 7 days. Media was then removed and replaced with Cell Titer Glo Luminescent Cell Viability Assay (Promega). Cells were incubated for 30 min at room temperature prior measurement of the relative light units (RLU). Data was normalized to the Vehicle condition.

### Statistical analyses

Results were analyzed using Prism Software (version 6, GraphPad, USA). Each sample is shown on each graph as an individual dot. Statistical significance was determined using one- or two-way ANOVA tests followed by Tukey or Sidak multiple comparisons tests to compare different groups with one or two variables respectively using a p< 0.05. The reported values are means ± SEM, as mentioned in relevant figure captions. Individual data points are shown on graphs when the samples size was lower than n< 10. Sample sizes, n, reported in figure legends. The statistical significance of the structural similarities between different viral polymerases were detected by FATCAT. FATCAT is evaluated by a P-value that measures the chance of getting the same similarity in two random structures. This P-value is calculated based on the empirical fitting of the extreme value distribution (EVD) to the FATCAT similarity score. The smaller the P-value, the more statistically significant the similarity between corresponding structures.

## Supporting information

S1 Fig Expression profile of human gene encoding for cellular factors of entry for SARS-CoV-2 virus. **a**. GTEx Transcriptome expression profile (log scale of TPM—transcripts per million) for the genes *ACE2*, *DDP4*, *BSG*, *TMPRSS2* and *NRP1* in different human tissues, including various brain regions. **b.** Various brain regions are color coded and correspond to quantification of each region in c. **c.** Expression of the genes *ACE2*, *DDP4*, *BSG*, *TMPRSS2* and *NRP1* in different brain regions and compartments from datasets of different repositories^45–47^. **d.** Gene expression levels of SARS-CoV-2 receptors in human BCO (in TPM). **e.** Receptor and cell surface marker gene expression in BCO (in TPM). Data was generated from bulk RNA sequencing data obtained from BCO. All data points are represented as individual points (n = 12 BCO). The raw data for the panels on this figure is located in S1 Data file.
(TIFF)

S2 Fig SARS-CoV-2 induces neuronal death. **a.** BCO immunostaining shows progenitor cells (SOX2^+^) neurons (MAP2^+^) and astrocyte (GFAP^+^ and AQP4^+^) expression. Scale bar upper panel 50 μm, lower panel 20 μm. Two different batches of BCOs from two different iPSC lines (WT83 and CVB) were used, and 5 organoids were analyzed per condition. **b.** Percentage of cell fractions showing NPC, neuron, and astrocyte populations within BCO. **c.** Upon BCO infection with SARS-CoV-2, we noted 477 differentially expressed genes at 1.25-fold change (p<0.05). Upregulated and downregulated genes (red and blue respectively) are represented (name of diagram, (-3, 3-fold change). Each sample represents three pooled organoids. **d.** Top 20 significant canonical pathways of the core analysis in IPA (Ingenuity) of most highly expressed genes in Mock vs SARS-CoV-2. p-values indicate the significance of enrichment for the most highly expressed genes from our dataset. Z-score > 0 in red, Z-score < 0 in blue, Z-score = 0 in white, Z-score unavailable in grey. **e.** The 477 differentially expressed genes at 1.25-fold change (p<0.05) were subjected to functional enrichment analyses using Metascape. Top 20 enriched pathways with their respective p-values are shown. **f.** Schematic of the experimental design; organoids BCO were first infected with SARS-CoV-2 and treated with Sofosbuvir (SOF) post-infection and analyzed for viral infection, synapse number and cell death 7 days after infection **g.** Quantification of SARS-CoV-2 intracellular mRNA by qPCR of BCO infected at MOI 2.5 and treated with vehicle (Veh) or increasing concentrations of SOF (1.25 μM, 3.2 μM, 8 μM, 20 μM, 50 μM). RNA was collected 7 days post-infection. Bars represent mean. Error bars represent SEM p**<0.01, ***p<0.001, n = 4 biological replicates (two pooled organoids derived from two independent batches from WT and CVB iPSCs per replicate, measured in triplicate). Significance was assessed using one-way ANOVA and Dunnett’s post-hoc test. **h.** Viability of BCO treated with vehicle (Veh) or increasing concentrations of SOF (1.28 μM, 3.2 μM, 8 μM, 20 μM and 50 μM). Viability was measured 7 days post-treatment. Relative light units (RLU) were normalized to VEH. Bars represent mean. Error bars represent SEM, n = 3 biological replicates (four pooled organoids per replicate). Significance was assessed using one-way ANOVA with Dunnett’s post-hoc test, no statistical difference was found. **i.** Western-blot of SARS-CoV-2 Nucleocapsid (N) infected BCO at MOI 2.5 treated with vehicle (Veh) or 20μM Sofosbuvir (SOF) 7 days post-infection. Beta actin was used as a loading control. n = 12 biological replicates (pooled into two protein lysates). The BCOs were fixed and analyzed 7 days post-infection. The raw data for the panels on this figure is located in S1 Data file.
(TIFF)

S3 Fig SARS-CoV-2 infects NPC, neuron and astrocytes. **a-d.** Immunolabeling of mock, infected, and infected, and SOF-treated organoid sections stained for TUNEL (white), SARS-CoV-2 N protein (green), Nestin (red), SOX2 (green), MAP2 (red), GFAP (red), respectively by confocal microscopy. Arrows point to colocalization with different cell types. Scale bar, 20 μm. These are representative images from the WT83 iPSC line.
(TIFF)

S4 Fig Characterization of neuronal populations impacted by SARS-CoV-2 infections within the organoids. Immunolabeling of mock and infected organoid sections stained for TUNEL (white) and excitatory upper (SATB2, CUX1), intermediate progenitors (TBR2), and lower cortical neuron markets (CTIP2). Images below each inset show split channels. Scale bar, 20 μm n = 5 biological replicates per condition. The BCOs were fixed and analyzed 7 days post-infection. These are representative images from the CVB iPSC line.
(TIFF)

S5 Fig Cell autonomous and non-cell autonomous impact of SARS-CoV-2 infections on neurons, synapses, and astrocytes. **a.** Immunolabeling SARS-CoV-2 infected organoids with MAP2 (red), SARS-CoV-2 (green) and TUNEL (white). **b.** Immunolabeling mock and SARS-CoV-2 infected organoids with MAP2 (red), SYN1 (green) and TUNEL (white). **c.** Immunolabeling mock and SARS-CoV-2 infected organoids with astrocytic markers Aquaporin-4 (AQP4, in red), GFAP (in white and green) and Vimentin (VIM in white). Images below each inset show split channels. Scale bar, 20 μm n = 5 biological replicates per condition. **d.** The integrated density was measured for each marker and normalized to mock-infected conditions and the total number of cells. Bars represent mean. Error bars represent SEM, n = 3 biological replicates. Significance was assessed using Students’ t-test, n.s. not significant. Two different batches of BCOs from two different iPSC lines (WT83 and CVB) were used, and 3 ROI from each of the 5 organoids were analyzed per condition. The BCOs were fixed and analyzed 7 days post-infection. The raw data for the panels on this figure is located in S1 Data file.
(TIFF)

S6 Fig SARS-CoV-2 decreases excitatory synapses, while influenza A does not induce cell death in BCO. **a.** The integrated density for SYN1 and PSD-95 was measured in mock-infected vehicle or SOF-treated organoids and normalized to mock-infected vehicle treated conditions and the total number of cells. Bars represent mean. Error bars represent SEM, n = 3 biological replicates. Significance was assessed using Students’ t-test, n.s. not significant. 3 ROIs per organoid, and 5 organoids per condition were assessed. **b.** Immunolabeling of vGLUT1-positive cells (yellow) within MAP2^+^ neurons (red) in BCO infected at MOI 2.5 and treated with vehicle (Veh) or 20μM Sofosbuvir (SOF). Scale bar, 20 μm. **c.** Immunolabeling of SYN1-positive cells (green) and PSD95 (red) within MAP2^+^ neurons (white) in BCO infected at MOI 2.5 and treated with vehicle (Veh) or 20μM Sofosbuvir (SOF). Scale bar, 20 μm. **d.** Immunolabeling of TUNEL (white), MAP2 (red), Influenza A virus nucleoprotein (NP, green) in BCO infected at MOI 2.5 and treated with Vehicle (Veh) or Sofosbuvir (SOF). Scale bar, 100 μm. Two different batches of BCOs from two different iPSC lines (WT83 and CVB) were used, and 5 organoids were analyzed per condition. The BCOs were fixed and analyzed 7 days post-infection. The panels a, b, c, and d are lower magnification images that are part of the figures shown and quantified in Fig 3**E.** Structural superposition of SARS-CoV-2 RdRp (colored blue) and Influenza A RdRp (colored yellow) shows minimal structural overlap within the polymerase active site. Both structures are not statistically similar (p = 5.20e-02), calculated from raw FATCAT score^73^. A total of 453 equivalent positions with an RMSD of 6.81Å and 5 twists were found between these two structures. **f-g.** Pairwise alignment of SARS-CoV-2 RdRP (nsp12) and HCV (NS5B) (d) and SARS-CoV-2 RdRP and Influenza A PB2 (e). Residues that partake in SOF binding or catalytic activity are highlighted in red to signify a match between proteins, orange to signify a partial match, and yellow to signify a mismatch. “*” = Residue identity is conserved; “.” = Residues have similar properties. The raw data for the panels on this figure is located on S1 Data file.
(TIFF)

S1 Video SARS-CoV-2 infects BCO. Immunolabeling of mock-infected organoid sections stained for TUNEL (white), SARS-CoV-2 N protein (green) and MAP2 (red) by confocal microscopy. Scale bar, 20 μm. The BCOs were fixed and analyzed 7 days post-infection.
(MOV)

S2 Video SARS-CoV-2 infects BCO. Immunolabeling of SARS-CoV-2 infected vehicle-treated organoid sections stained for TUNEL (white), SARS-CoV-2 N protein (green), and MAP2 (red) by confocal microscopy. Scale bar, 20 μm. The BCOs were fixed and analyzed 7 days post-infection.
(MOV)

S3 Video SARS-CoV-2 infects BCO. Immunolabeling of SARS-CoV-2 infected SOF-treated organoid sections stained for TUNEL (white), SARS-CoV-2 N protein (green), and MAP2 (red) by confocal microscopy. Scale bar, 20 μm. The BCOs were fixed and analyzed 7 days post-infection.
(MOV)

S1 Table List of differentially expressed genes in the bulk RNA-sequencing in mock vs SARS-CoV-2 infected BCO. A total of 477 genes are differentially expressed between mock and SARS-CoV-2 infected BCO (fold change 1.25, p value <0.05). The BCOs were analyzed 7 days post-infection.
(XLSX)

S2 Table PANTHER analyses of RNA sequencing obtained through Rosalind Software. The BCOs were analyzed 7 days post-infection.
(XLSX)

S3 Table Bulk RNA sequencing information. (XLSX)

S1 Data Raw data related to each individual figure. (XLSX)

S1 Raw Images Original, raw, and uncropped images of the Western Blots presented in Figs 1G and S2I. (PDF)
