# Supplementary figures and images for: SARS-CoV-2 infects human brain organoids causing cell death and loss of synapses that can be rescued by treatment with Sofosbuvir
Source: PLoS Biol. 2022 Nov 3;20(11):e3001845. doi: 10.1371/journal.pbio.3001845 (PMC9632769; doi:10.1371/journal.pbio.3001845)

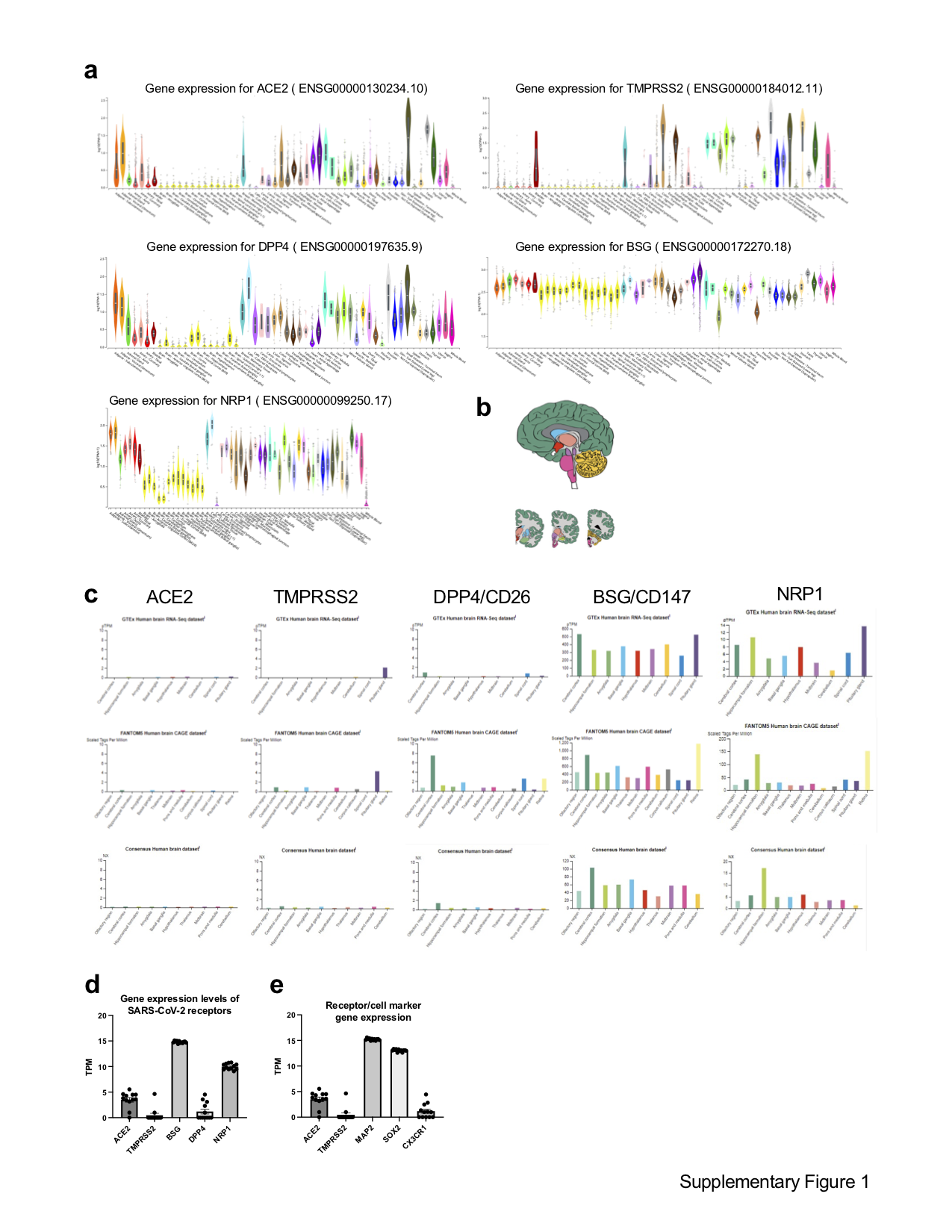

Supplement: S1 Fig — a. GTEx Transcriptome expression profile (log scale of TPM—transcripts per million) for the genes ACE2, DDP4, BSG, TMPRSS2 and NRP1 in different human tissues, including various brain regions. b. Various brain regions are color coded and correspond to quantification of each region in c. c. Expression of the genes ACE2, DDP4, BSG, TMPRSS2 and NRP1 in different brain regions and compartments from datasets of different repositories45–47. d. Gene expression levels of SARS-CoV-2 receptors in human BCO (in TPM). e. Receptor and cell surface marker gene expression in BCO (in TPM). Data was generated from bulk RNA sequencing data obtained from BCO. All data points are represented as individual points (n = 12 BCO). The raw data for the panels on this figure is located in S1 Data file. (TIFF) [file pbio.3001845.s001.tiff]

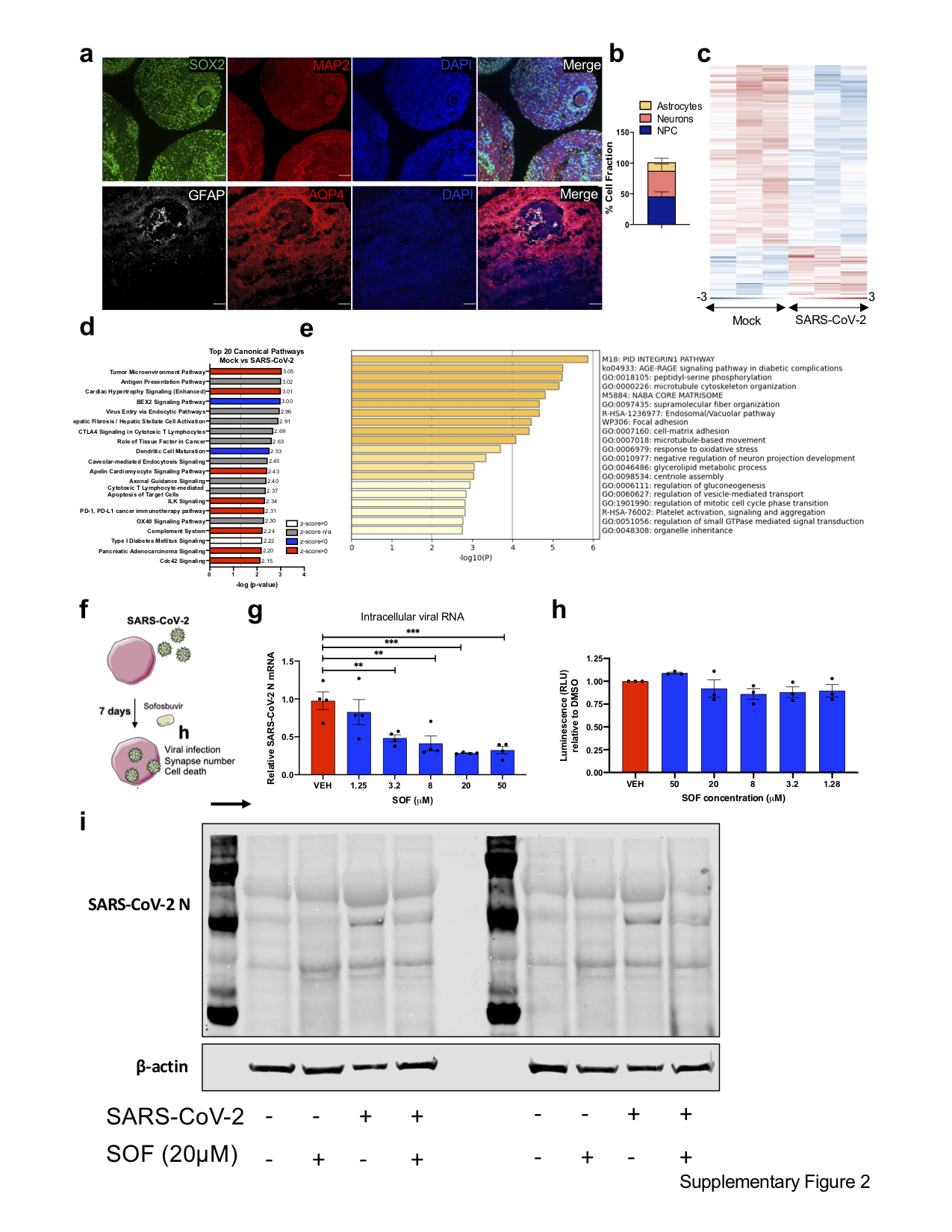

Supplement: S2 Fig — a. BCO immunostaining shows progenitor cells (SOX2+) neurons (MAP2+) and astrocyte (GFAP+ and AQP4+) expression. Scale bar upper panel 50 μm, lower panel 20 μm. Two different batches of BCOs from two different iPSC lines (WT83 and CVB) were used, and 5 organoids were analyzed per condition. b. Percentage of cell fractions showing NPC, neuron, and astrocyte populations within BCO. c. Upon BCO infection with SARS-CoV-2, we noted 477 differentially expressed genes at 1.25-fold change (p<0.05). Upregulated and downregulated genes (red and blue respectively) are represented (name of diagram, (-3, 3-fold change). Each sample represents three pooled organoids. d. Top 20 significant canonical pathways of the core analysis in IPA (Ingenuity) of most highly expressed genes in Mock vs SARS-CoV-2. p-values indicate the significance of enrichment for the most highly expressed genes from our dataset. Z-score > 0 in red, Z-score < 0 in blue, Z-score = 0 in white, Z-score unavailable in grey. e. The 477 differentially expressed genes at 1.25-fold change (p<0.05) were subjected to functional enrichment analyses using Metascape. Top 20 enriched pathways with their respective p-values are shown. f. Schematic of the experimental design; organoids BCO were first infected with SARS-CoV-2 and treated with Sofosbuvir (SOF) post-infection and analyzed for viral infection, synapse number and cell death 7 days after infection g. Quantification of SARS-CoV-2 intracellular mRNA by qPCR of BCO infected at MOI 2.5 and treated with vehicle (Veh) or increasing concentrations of SOF (1.25 μM, 3.2 μM, 8 μM, 20 μM, 50 μM). RNA was collected 7 days post-infection. Bars represent mean. Error bars represent SEM p**<0.01, ***p<0.001, n = 4 biological replicates (two pooled organoids derived from two independent batches from WT and CVB iPSCs per replicate, measured in triplicate). Significance was assessed using one-way ANOVA and Dunnett’s post-hoc test. h. Viability of BCO treated with vehicle (Veh) or in [file pbio.3001845.s002.tiff]

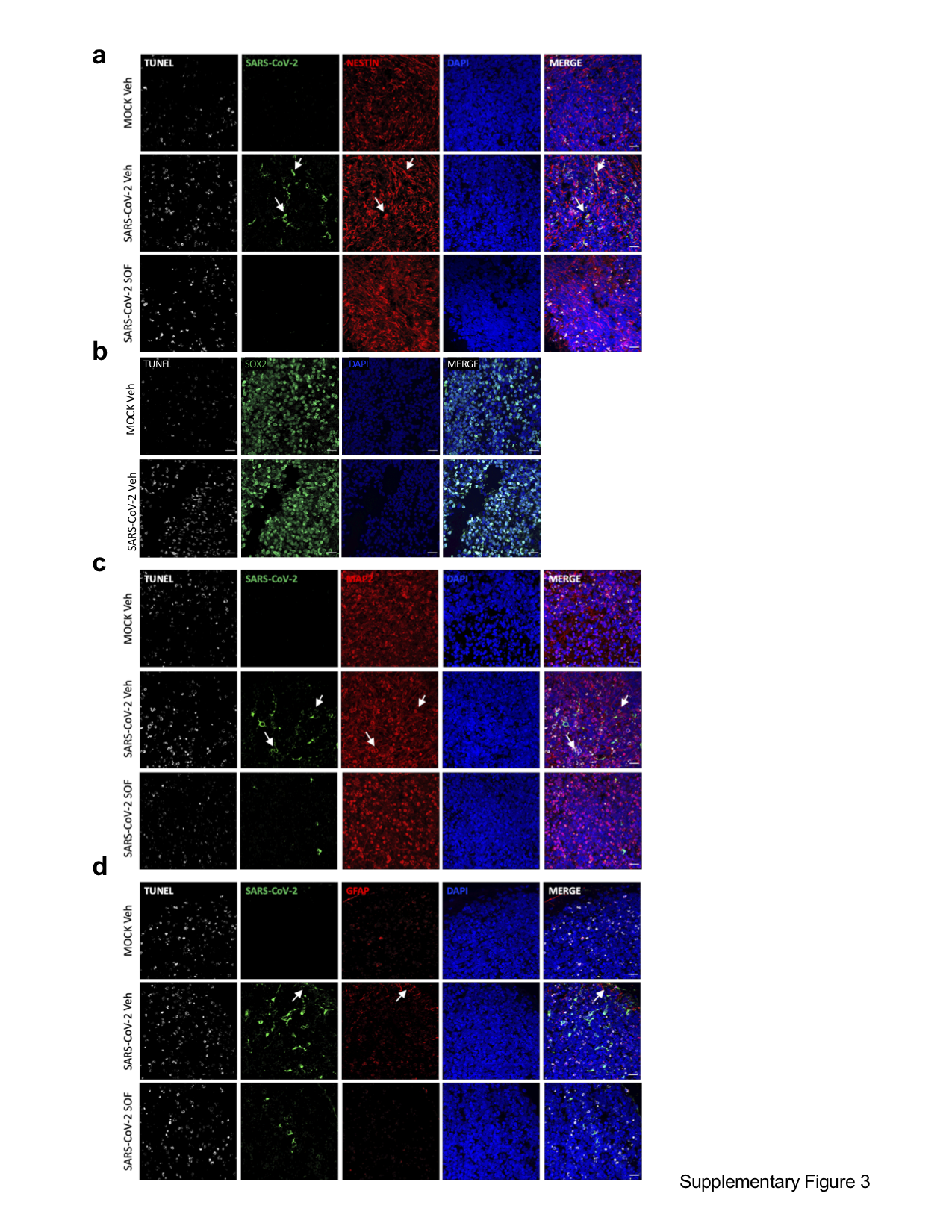

Supplement: S3 Fig — a-d. Immunolabeling of mock, infected, and infected, and SOF-treated organoid sections stained for TUNEL (white), SARS-CoV-2 N protein (green), Nestin (red), SOX2 (green), MAP2 (red), GFAP (red), respectively by confocal microscopy. Arrows point to colocalization with different cell types. Scale bar, 20 μm. These are representative images from the WT83 iPSC line. (TIFF) [file pbio.3001845.s003.tiff]

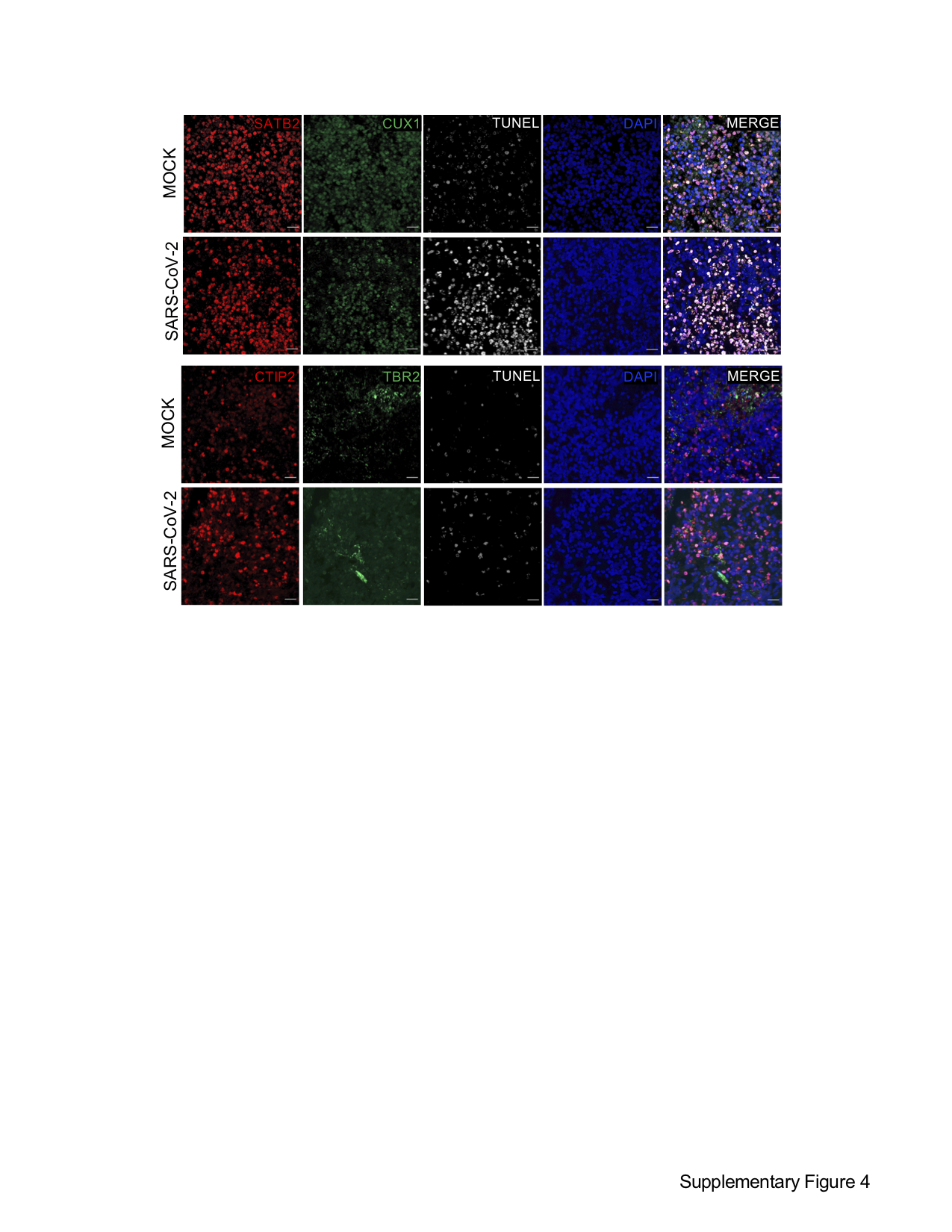

Supplement: S4 Fig — Immunolabeling of mock and infected organoid sections stained for TUNEL (white) and excitatory upper (SATB2, CUX1), intermediate progenitors (TBR2), and lower cortical neuron markets (CTIP2). Images below each inset show split channels. Scale bar, 20 μm n = 5 biological replicates per condition. The BCOs were fixed and analyzed 7 days post-infection. These are representative images from the CVB iPSC line. (TIFF) [file pbio.3001845.s004.tiff]

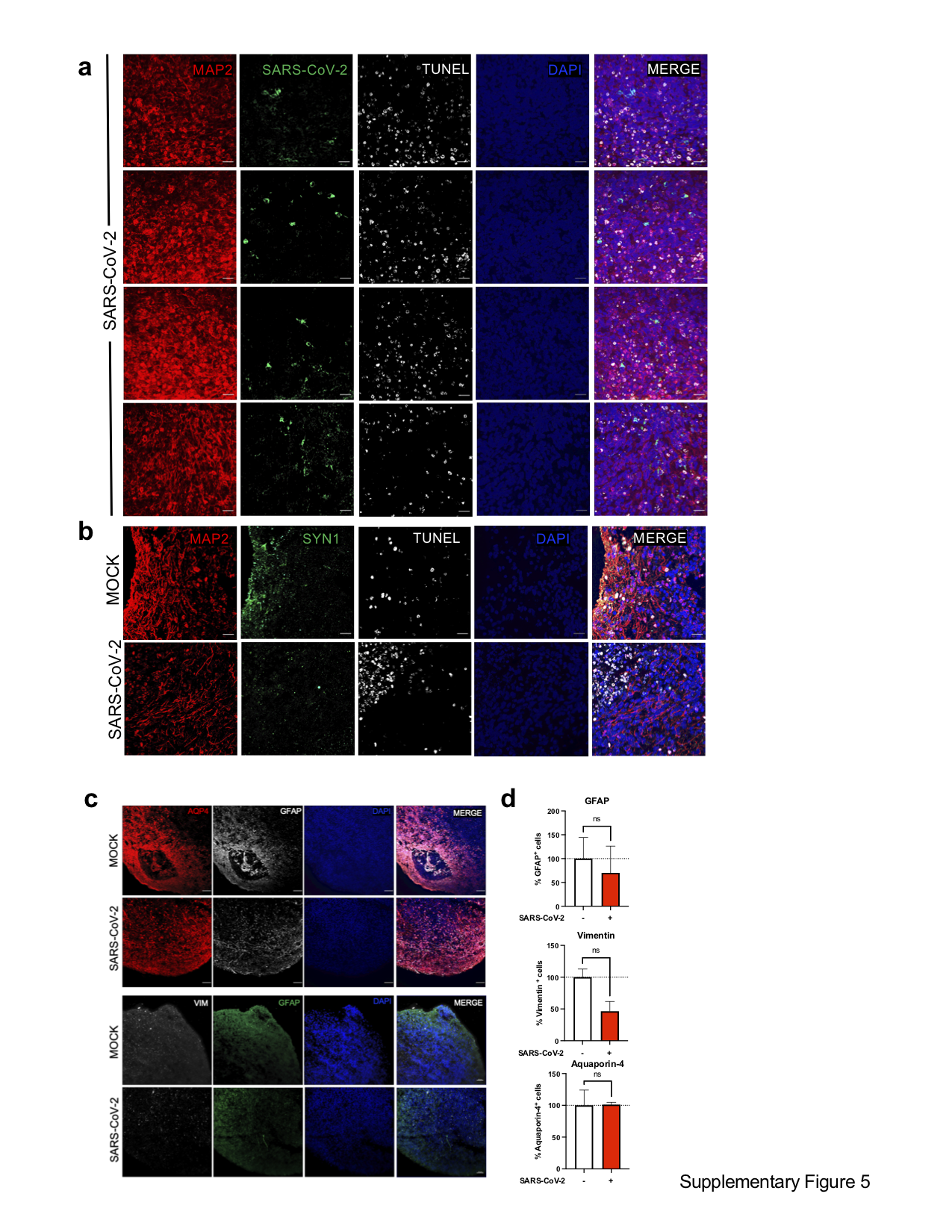

Supplement: S5 Fig — a. Immunolabeling SARS-CoV-2 infected organoids with MAP2 (red), SARS-CoV-2 (green) and TUNEL (white). b. Immunolabeling mock and SARS-CoV-2 infected organoids with MAP2 (red), SYN1 (green) and TUNEL (white). c. Immunolabeling mock and SARS-CoV-2 infected organoids with astrocytic markers Aquaporin-4 (AQP4, in red), GFAP (in white and green) and Vimentin (VIM in white). Images below each inset show split channels. Scale bar, 20 μm n = 5 biological replicates per condition. d. The integrated density was measured for each marker and normalized to mock-infected conditions and the total number of cells. Bars represent mean. Error bars represent SEM, n = 3 biological replicates. Significance was assessed using Students’ t-test, n.s. not significant. Two different batches of BCOs from two different iPSC lines (WT83 and CVB) were used, and 3 ROI from each of the 5 organoids were analyzed per condition. The BCOs were fixed and analyzed 7 days post-infection. The raw data for the panels on this figure is located in S1 Data file. (TIFF) [file pbio.3001845.s005.tiff]

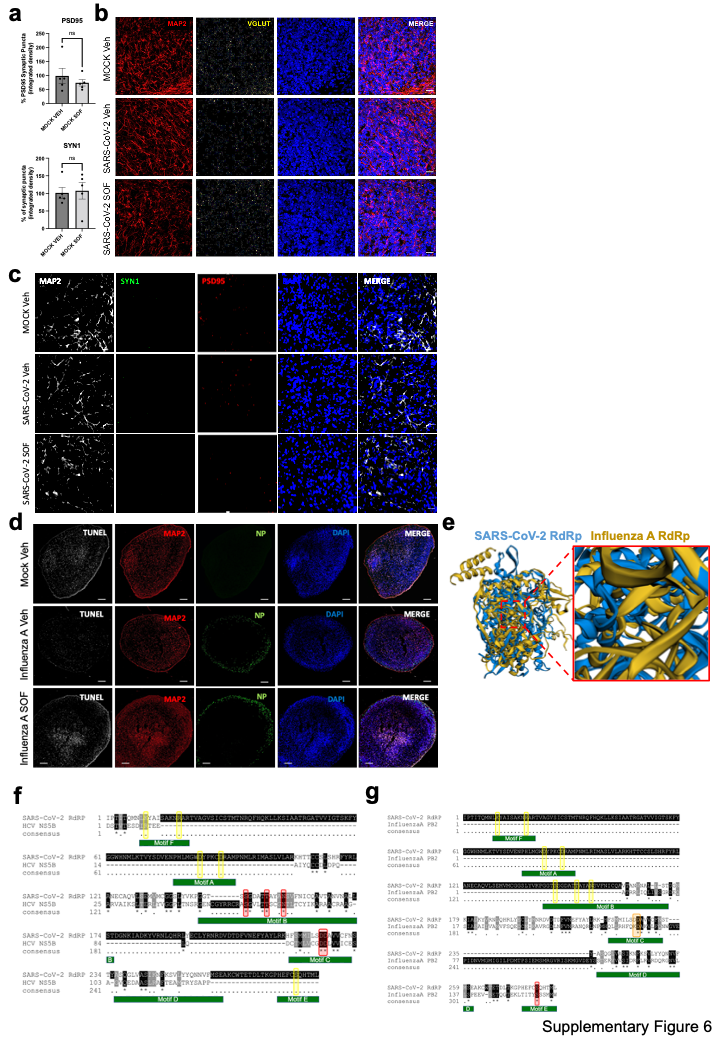

Supplement: S6 Fig — a. The integrated density for SYN1 and PSD-95 was measured in mock-infected vehicle or SOF-treated organoids and normalized to mock-infected vehicle treated conditions and the total number of cells. Bars represent mean. Error bars represent SEM, n = 3 biological replicates. Significance was assessed using Students’ t-test, n.s. not significant. 3 ROIs per organoid, and 5 organoids per condition were assessed. b. Immunolabeling of vGLUT1-positive cells (yellow) within MAP2+ neurons (red) in BCO infected at MOI 2.5 and treated with vehicle (Veh) or 20μM Sofosbuvir (SOF). Scale bar, 20 μm. c. Immunolabeling of SYN1-positive cells (green) and PSD95 (red) within MAP2+ neurons (white) in BCO infected at MOI 2.5 and treated with vehicle (Veh) or 20μM Sofosbuvir (SOF). Scale bar, 20 μm. d. Immunolabeling of TUNEL (white), MAP2 (red), Influenza A virus nucleoprotein (NP, green) in BCO infected at MOI 2.5 and treated with Vehicle (Veh) or Sofosbuvir (SOF). Scale bar, 100 μm. Two different batches of BCOs from two different iPSC lines (WT83 and CVB) were used, and 5 organoids were analyzed per condition. The BCOs were fixed and analyzed 7 days post-infection. The panels a, b, c, and d are lower magnification images that are part of the figures shown and quantified in Fig 3E. Structural superposition of SARS-CoV-2 RdRp (colored blue) and Influenza A RdRp (colored yellow) shows minimal structural overlap within the polymerase active site. Both structures are not statistically similar (p = 5.20e-02), calculated from raw FATCAT score73. A total of 453 equivalent positions with an RMSD of 6.81Å and 5 twists were found between these two structures. f-g. Pairwise alignment of SARS-CoV-2 RdRP (nsp12) and HCV (NS5B) (d) and SARS-CoV-2 RdRP and Influenza A PB2 (e). Residues that partake in SOF binding or catalytic activity are highlighted in red to signify a match between proteins, orange to signify a partial match, and yellow to signify a mismatch. “*” = Residue identity is conserved; [file pbio.3001845.s006.tiff]

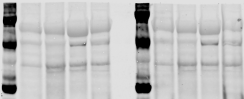

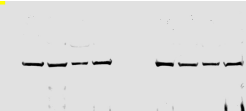

Supplement: S1 Raw Images — (PDF) [file pbio.3001845.s014.pdf]
